# Unraveling atomic complexity from frozen samples

**DOI:** 10.1063/4.0000303

**Published:** 2025-04-18

**Authors:** Yasmeen N. Ruma, Brent L. Nannenga, Tamir Gonen

**Affiliations:** 1 Department of Biological Chemistry, University of California, Los Angeles, Los Angeles, California 90095, USA; 2 Howard Hughes Medical Institute, University of California, Los Angeles, Los Angeles, California 90095, USA; 3 Chemical Engineering, School for Engineering of Matter, Transport and Energy, Arizona State University, Tempe, Arizona 85287, USA; 4 Center for Applied Structural Discovery, Biodesign Institute, Arizona State University, Tempe, Arizona 85281, USA; 5 Department of Physiology, University of California, Los Angeles, Los Angeles, California 90095, USA

## Abstract

Cryo-electron microscopy (cryo-EM) is a significant driver of recent advances in structural biology. Cryo-EM is comprised of several distinct and complementary methods, which include single particle analysis, cryo-electron tomography, and microcrystal electron diffraction. In this Perspective, we will briefly discuss the different branches of cryo-EM in structural biology and the current challenges in these areas.

## INTRODUCTION

Structural biology has significantly evolved over the past several decades. The ability to determine structures of biological macromolecules, specifically proteins and nucleic acids, at high resolutions has provided answers to questions regarding their function. This has shed light on how these biomolecules are involved in the formation of complexes, their arrangement in cells, whether they are potential drug targets, the ligand binding sites of important target molecules, the impact of mutations and structural modifications on their function, and several other pertinent questions. To date, the number of structures deposited in Protein Data Bank (PDB) is 220 000 (https://www.rcsb.org/search\). Two of the major areas of high-resolution biomolecular structure determination are x-ray crystallography and cryo-electron microscopy (cryo-EM).

In the past, x-ray crystallography has dominated the field of structural biology as the primary technique for determining high-resolution protein structures.^1^ X ray crystallography involves crystallizing the purified protein of interest and obtaining x-ray diffraction data on these large, well-ordered protein crystals. While x-ray crystallography remains a powerful tool for structural biology, bottlenecks associated with crystallizing difficult targets, such as membrane proteins and large macromolecular complexes, remain prevalent. Therefore, significant efforts aimed at improving crystallography and developing complementary structure determination methods began decades ago.

One such area that enjoyed significant advancement and success is the field of cryo-EM. Cryo-EM has emerged from its dark ages as a boutique technique known for “blobology”^2^ to a powerful approach with several complementary modalities to determine atomic resolution structures. Cryo-EM is based on the general approach of exposing samples to an electron beam in a transmission electron microscope (TEM) under cryogenic conditions, which can preserve their hydrated state within the high vacuum of the TEM and help reduce the effects of radiation damage.^3^

The groundwork for cryo-EM began in the 20th century to study protein structure using transmission electron microscopes. As early as 1937, images of catalase crystals were recorded.^4^ In 1968, DeRosier and Klug first demonstrated that three-dimensional structures can be generated from two-dimensional images captured on an electron microscope. They developed a 3D reconstruction method to determine the structure of the extended tail of the T4 bacteriophage from Fourier transforms of electron microscope images.^5^ Later, Matricardi *et al.*^6^ demonstrated the collection of high-resolution electron diffraction from hydrated catalase crystals using a hydration stage. Taylor and Glaeser showed that high-resolution diffraction data could be obtained by freezing catalase crystals as they were embedded in sugar, thus preserving the native hydrated state.^7^ In 1975, Henderson and Unwin published the first 3D structural model of glucose-embedded 2D crystals of the purple membrane protein bacteriorhodopsin and bovine liver catalase at 7 and 9 Å resolutions, respectively,^8,9^ by combining the intensities from electron diffraction with the phases from electron microscopy images. Later, with the development of the plunge freezing technique in 1984 by Dubochet and co-workers, it became possible to rapidly freeze and vitrify biological samples in their native hydrated state.^10^

Since then, several protein structures have been solved to near atomic resolution. The first few cryo-EM structures were derived from the electron diffraction and imaging of two-dimensional protein crystals, which include the structure of purple membrane from *Halobacterium halobium*,^11^ the alpha beta tubulin dimer,^12^ and aquaporins.^13–15^ Meanwhile, significant progress was made in determining the structures of single particles of viruses,^10,16^ ribosomes,^17^ and membrane channels^18^ by imaging and reconstructing the particles, an approach now known as single particle analysis (SPA). By the late 2000s, there was a significant improvement in the resolution of SPA cryo-EM structures from subnanometer to near atomic resolution. The first SPA structures at a resolution of ∼4 Å were reported in 2008, and subsequently, more structures at this resolution became achievable.

Many of the advances in cryo-EM can be attributed to faster and more sensitive cameras, such as direct electron detectors^19^ coupled to automated data collection processes, and better image reconstruction software and hardware. Autoloaders, continuously powered lenses, more stable sample stages, and aberration-free image shifting are all advanced features that have made it easier to automate cryo-electron microscopes. Moreover, the processing of data has become more convenient for users, as automated pipelines are now being created to make the acquisition and processing of data more efficient^20^ thus requiring less expert knowledge.^21^ In addition, the use of energy filters has become more commonplace, which is focused on reducing the energy spread of incoming electrons, thereby improving the signal in the micrographs. ^20,22^With all these technological advances in electron microscopy, the field entered the “resolution revolution,”^23^ and evolved into a disruptive technology to unravel the structure of several samples that were not attainable by x-ray crystallography.

Today, cryo-EM is widely used in structure determination and is not just confined to protein structures.^24^ Single particle analysis (SPA),^25^ cryo-electron tomography (cryo-ET),^26^ and MicroED^27^ are branches of cryo-EM each entailing its own strengths and possibilities for improvement and further development. As of May 2024, more than 35 000 entries of structures were deposited to the electron microscopy data bank (EMDB).^28^ Although cryo-EM is promising, there are still challenges that need to be overcome.^29^ Several reviews are available on the various modalities of cryo-EM^24,27,30,31^ and in this perspective, we will give an overview of the different cryo-EM methods and inject some of our opinions.

## SINGLE PARTICLE ANALYSIS (SPA)

**FIG. 1.**
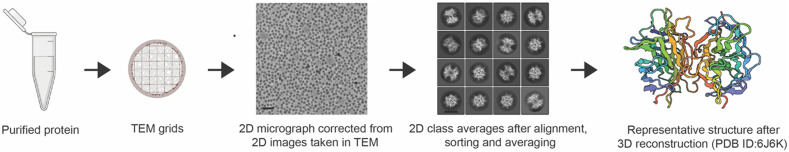
Workflow of cryo-EM single particle analysis. The images of the micrographs were used from Fan *et al.,* Nat. Commun. **10**, 2386 (2019).^47^ Copyright 2019 Authors, a Creative Commons Attribution (CC BY) license.

In the last 20 years, SPA has become a valuable tool in structural biology for studying complex, flexible, or highly dynamic proteins as it does not involve the need for crystallization and generally requires less purified protein.^32^ Here, protein samples are exposed to electrons in a TEM, and the projection images are compiled to generate a 3D structure (Fig. 1).

To determine a protein structure by SPA, purified protein samples are applied to electron microscopy grids made of a thin layer of holey carbon film, followed by blotting to remove excess solution, leaving a thin film of sample suspended in the holes of the grid. The grid is then vitrified by plunging into liquid ethane. Ideally, the protein molecules are distributed in random orientation in the grid holes. The sample is then imaged in the cryo-TEM, and thousands of projection images are captured. Because direct electron detectors are currently used almost exclusively for SPA, images are recorded as movies with a high frame rate so that motion correction can be used to unblur the images, leading to higher resolutions.^20,33,34^ Particles in the same orientation are classified in 2D and averaged using alignment algorithms to improve the signal to noise ratio. Poor particles are also filtered out at this stage, ensuring a high quality stack of particles for structure determination. The curated stacks of single particles are then used to reconstruct the 3D model.^35^ The software packages commonly used to process single particle images are cryoSPARC,^36^ Relion,^37^ cisTEM,^38^ SPHIRE,^30^ and EMAN2.^39^ Software based on deep learning used for SPA includes crYOLO,^40^ DeepEM,^41^ TOPAZ,^42^ Deep Picker^43^ for particle picking, and cryoDRGN^44^ for identification and classification of particles. Advances in data collection and image processing software have allowed scientists to determine structures with SPA that can rival the resolutions produced by crystallographic methods.^45,46^

SPA has proven to be a useful method in determining the structure of large integral membrane proteins, which were not attainable by x-ray crystallography.^30^ Moreover, SPA can also deliver information about protein dynamics if a sufficient number of particles are imaged and classified into different states.^30^ For example, the structure of the transient receptor potential (TRP) ion channel was unresolved for years, but with the application of SPA, Liao *et al.* were able to determine the structure of TRPV1 (one of the members of the TRP superfamily) in three different functional states.^48^ Examples of important membrane protein structures determined by SPA include voltage‐gated calcium channel Ca_v_1.1,^49^ sodium channel Na_v_,^50^ ATP‐binding cassette subfamily G member 2 (ABCG2)^51^ and membrane protein complex, such as a class B G protein‐coupled receptor (GPCR)–G protein complex.^52^ The most recent example of a membrane protein complex is the structure of *Trypanosoma brucei* water channel aquaglyceroporin 2 (TbAQP2), in complex with the anti-trypanosomatid drug, pentamidine,^53^ needed to prevent African sleeping sickness. In addition to membrane proteins, single particle cryo-EM was also successfully used to determine the first high-resolution structures of tau filaments isolated from the brain of a patient with Alzheimer's disease,^54^ amyloid-β(1–42) fibrils,^55^ and microtubules in complex with various anticancer compounds, a potential for drug discovery.^56^

Although SPA has undoubtedly provided major advancements for structural biologists, there are still obstacles to overcome in determining structures of difficult targets, especially small membrane proteins. Like many proteins, membrane proteins may be in continuous motion during biochemical reactions in the cell and so when extracted, they may exist in different conformations and exhibit conformational heterogeneity. This complicates the use of SPA to solve the structures of proteins that are flexible and highly heterogenous.^29^ Techniques such as normal mode analysis^57^ and manifold embedding approach^58^ have been used to solve the flexibility issue. Very recently, a deep learning algorithm known as 3DFlex was developed to improve the resolution of flexible proteins.^59,60^ However, because SPA relies on imaging individual particles, the smaller the particle the weaker the signal. Although the calculated physical limitation of SPA imaging is estimated at 20–40 kDa^61,62^ it is yet to be achieved. This perhaps presents the largest current obstacle for cryo-EM: the majority of the human proteome consists of proteins that are far smaller, as the average size of a protein in a human cell is 35 kDa.^63^ As imaging targets less than 100 kDa is very currently challenging and the resolutions are lower than for larger targets, many proteins currently remain purely in the realm of crystal based techniques for high-resolution structural studies.^64^

The size restriction is a major drawback when studying small membrane proteins. Most membrane proteins are very small, less than 40 kDa, with many signaling type 1 and type 2 membrane proteins even smaller. These proteins are typically extracted with detergent, where the detergent can mask the signal from these small proteins.^65,66^ Several approaches, such as amphipols,^67^ bicelles,^68^ nanodiscs,^69^ proteolipososmes,^70^ etc., were used as a substitute to detergents to maintain the stability of protein and aid in structure determination; however, the small protein size still presents a formidable challenge.

Several ways to overcome the size limitation in SPA have been documented, including forming larger complexes, for example, between protein antigen-binding fragments (Fabs),^71^ green fluorescent protein (GFP),^72^ maltose binding protein (MBP),^73^ and a modular adaptor protein, designed ankyrin repeat proteins (DARPins).^72^ Another approach is using scaffolding proteins to specifically recognize and bind target proteins and increase their mass.^73^ While these approaches are viable, they come at a huge cost, and the upstream biochemical sample preparation and screening are similar to crystallographic preparations.

## CRYO-ELECTRON TOMOGRAPHY

**FIG. 2.**
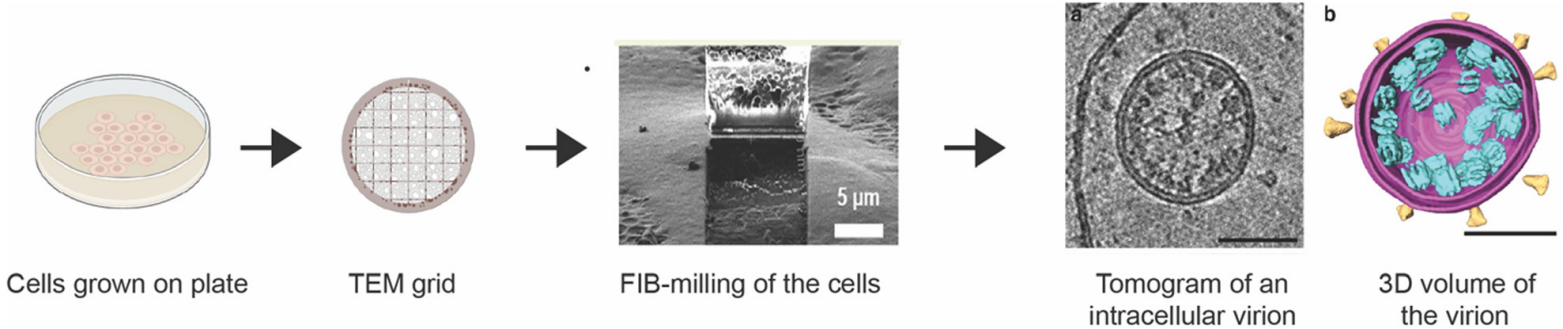
Workflow of cryo-ET. The image of the lamellae and the tomogram were adapted from Li *et al.*, Commun. Biol. **6**, 474 (2023)^78^ and Klein *et al.*, Nat. Commun. **11**, 5885 (2020),^79^ respectively. Copyright (2020) Authors, a Creative Commons Attribution (CC BY) license.

SPA requires the extraction of protein from its native cellular environment, which can hinder their association with membranes, binding partners in the cell, and environment-specific interactions and effects.^24^ Therefore, an effective way to understand the structure of macromolecules, their interactions in the cell, and the overall cellular structure, is to determine structures directly within the cells. Cryo-electron tomography (cryo-ET) is a technique used to image native cells, tissues, or small organisms in the electron microscope by preserving the cellular contents in their native state.^74^ Instead of purified protein, here cells or tissues of interest are frozen on EM grids and imaged (Fig. 2).

For many samples, the thickness of the cells is limiting in cryo-ET, as imaging in TEM requires the thickness of sample to be below ∼300 nm,^74^ so that electrons can penetrate through the material. This limitation was recently addressed by the use of cryo-focused ion beam milling (cryo-FIB). This is carried out in a dual beam focused ion beam–scanning electron microscope (FIB–SEM), where a beam of focused ions (typically gallium) is used to remove material until the desired thickness is achieved.^75^ An additional advancement in the use of cryo-FIB milling for cellular tomography has been the addition of correlative light EM (cryo-CLEM).^76^ Cryo-CLEM is used to locate the desired cellular structures or a fluorescently labeled molecule in the sample using light microscopy, and these areas are correlated within the FIB-SEM for milling at these locations.^77^

Once the lamella of the desired thickness is obtained, images are acquired as a tilt series in the TEM. These images are then combined to reconstruct a volume of the sample and provide three-dimensional (3D) data known as cryo-electron tomograms. These data can then be used to reconstruct the cellular environment and, in the case of identifiable macromolecules, determine macromolecular structure by subtomogram averaging.^74^ There are several software packages used in cryo-ET data processing for the complete pipeline, such as Warp,^80^ M,^81^ IMOD,^82^ STOPGAP,^83^ tomoDRGN,^84^ Relion,^85^ emClarity,^86^ EMAN2,^87^ Dynamo package,^88^ tomoBEAR,^89^ AreTomo,^90^ nextPYP,^91^ IsoNet,^92^ and Amira (ThermoFisher scientific). To date, EMDB has 2144 entries of macromolecular structures determined by subtomogram averaging. The structure with the highest resolution, mouse heavy chain apoferritin^91^ (1.8 Å), was determined by subtomogram averaging.

Cryo-ET is emerging as a powerful technique due to its ability to determine structures within the cell, including the calcium channel CatSper in sperm flagella,^93^ coronavirus 2 (SARS-CoV-2) spike protein during intracellular infection,^79^ and the potential to map the structural heterogeneity inside cells (e.g., the study of proteasome location in algae^94^). Additionally, cryo-ET is commonly used to determine ultrastructural features of the cellular environment itself. For example, Collado *et al.*^95^ identified that the protein tricalbin forms the endoplasmic reticulum curvature to maintain plasma membrane integrity.^74^

However, there are some drawbacks to this method. The major current bottleneck within the field of cryo-ET is the low-throughput workflow and the requirement for a suite of advanced instrumentation beyond the cryo-TEM (e.g., cryo-CLEM systems and cryo-FIB-SEMs). In addition, imaging the same region during the collection of tomograms adds significantly more radiation damage in cryo-ET relative to other cryo-EM modalities,^96^ which in turn results in lower overall attainable resolutions. Overall, cryo-ET still requires a high level of expertise, and further development must follow. Furthermore, the high-end instrumentation required represents a large barrier to many groups; therefore, additional support for user-facilities and equipment is needed.

## MICROCRYSTAL ELECTRON DIFFRACTION

**FIG. 3.**
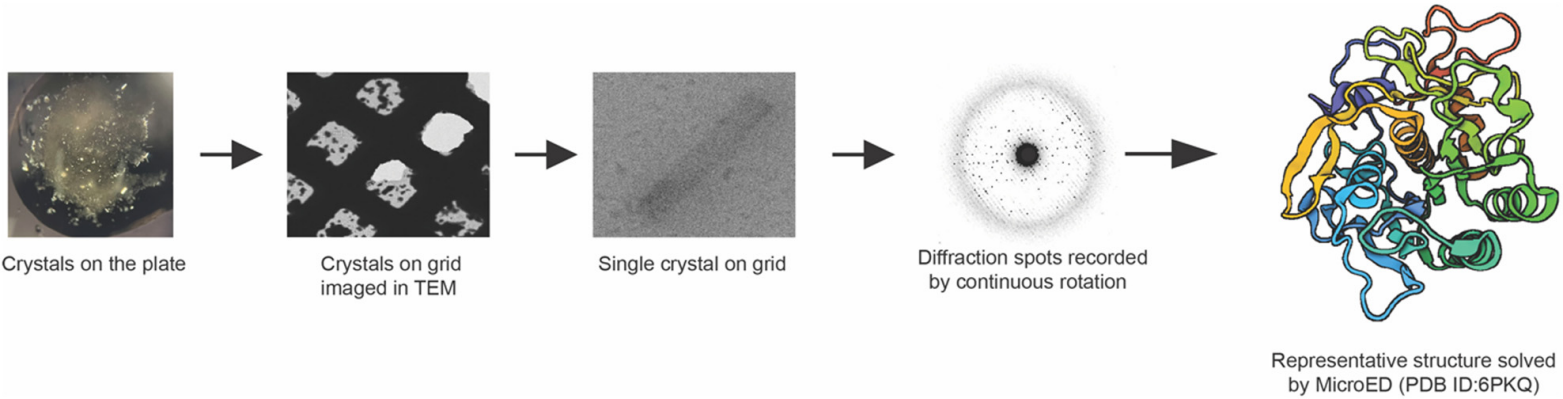
Workflow of MicroED.

While both single particle analysis and tomography rely on imaging, the cryo-EM method, MicroED, employs diffraction within the cryo-TEM. In MicroED (Fig. 3), ultra-low electron doses are used to diffract small three-dimensional micro- and nanocrystals in a TEM.^27^ Since its introduction in 2013, MicroED has grown continuously and has become a formidable branch of cryo-EM pushing its boundaries to determine the structures of not only macromolecules but also small organic and inorganic molecules, peptides, natural products, and pharmaceuticals.^97^ A major advantage of MicroED over x-ray crystallography is that it can be used to collect data from crystals a billionth the size of those needed for that of conventional x-ray diffraction.^98–101^

The workflow of MicroED involves growing crystals by approaches that are similar to what is used for x-ray crystallography.^102^ Small micro- and nanocrystals are harvested, applied to the surface of an EM grid and vitrified. Crystal quality is assessed in the cryo-TEM by collecting single electron diffraction pattern. When quality crystals are identified, full continuous rotation electron diffraction datasets are collected from each crystal.^103^ As the crystal is continuously rotated in the beam, MicroED data are collected using a fast camera as a movie. Each frame in that movie corresponds to an integrated wedge of diffraction data in reciprocal space.^104,105^ The structure of the target molecule is then elucidated through data processing and refinement workflows that consist of common x-ray data processing software, such as XDS^106^ for data processing, Phaser for molecular replacement,^107^ Phenix.refine^108^ for structure refinement, and Coot for visualization and model building.^109^ In the case of crystals that are too thick for the electron beam, cryo-FIB milling is employed to mill the crystals to produce a thin lamella^110,111^ with ideal thickness optimized for MicroED.^112^

Reliable structure determination using electron diffraction from 3D crystals was considered impossible for decades.^113^ The main concerns had to do with dynamical scattering.^114^ Several laboratories investigated the use of 3D macromolecular crystals in electron diffraction and concluded that dynamical scattering resulted in random intensities that invalidated the approach.^115,116^ Computer simulations suggested that if crystals were thicker than a mere 100 nm, the dynamical effects would already be severe enough to yield random intensities.^117^ Several approaches were developed also in the materials science domain, for example, precession^118^ and rotation electron diffraction, to overcome these problems. However, these approaches are not suitable for macromolecules because they require significantly larger doses than most macromolecules can withstand. In 2013, it was demonstrated that dynamical scattering does not prohibit structure determination when electron diffraction is collected as a series,^119^ and indeed later with continuous rotation^103^ the quality of the data was vastly improved.

The first novel structure determined by continuous-rotation MicroED was that of α-synuclein NACore, the segment responsible for amyloid formation,^120^ which had resisted structure determination efforts for many years prior to the application of MicroED. Later in 2018, the structure of the prototypical tetrameric Na^+^-conducting channel, NaK, was identified in two conformations at 2.5 Å, one of which was previously unobserved—a new transient state was identified in which a hydrated Na^+^ ion was spotted at the entrance of the channel pore, demonstrating MicroED's ability to study membrane protein dynamics.^121^ The functional mutant of the voltage dependent anion channel (VDAC) from mitochondria,^122^ the MyD88^123^ and protoglobin^124^ are additional examples of novel structures that could not be determined by x-ray crystallographic approaches and were too small for SPA. MicroED has also proved effective to determine structures of GPCRs without bound G proteins. This was first demonstrated on the adenosine A2a receptor A_2A_AR,^125^ and later the same approach was used to determine the structure of the vasopressin 1b receptor, V1bR,^126^ which had resisted other structural biology methods despite major efforts. The more recent novel structure determined by MicroED is that of the tight junction forming lens membrane protein, MP20.^127^ This protein is a small, 18 kDa membrane protein that traverses the membrane four times and resisted structure determination for decades.

MicroED is also routinely used to determine high-resolution structures of small molecules,^128,131^ materials, natural products, and drugs.^129,130^ This is achieved by applying the sample as an amorphous powder or suspension of small molecule crystals on the surface of holey carbon EM grids. Electron diffraction data are collected using similar procedures described above.^131^ Here, the experiment can be done either at room temperature or in cryogenic conditions; however, cryogenic conditions are always recommended to reduce the effects of radiation damage and lead to higher attainable resolutions especially for beam sensitive samples.^132^ Examples of small molecule drugs include carbamazepine,^133^ the antihistamine meclizine hydrochloride,^129^ mirabegron in two distinct conformations,^134^ and the macrocyclic drugs simeprevir^135^ and paritaprevir^130^ used for treating chronic hepatitis C virus (HCV) infection. Natural products solved by MicroED include 3-methyloxindole and 1-methyl-2-indanone.^136–138^

While MicroED is fast becoming indispensable in the pharmaceutical and drug discovery industries,^137^ challenges still persist that require substantial development and optimization. For example, while several novel macromolecular structures were recently reported (see above), sample preparation remains difficult and requires a high level of expertise.^139^ While for SPA and tomography, samples are added directly from the solution, for crystals the sample preparation process is a lot more delicate. Too much blotting, and the crystal lattice could collapse; too little blotting and the sample becomes too thick and the electron beam is unable to penetrate. Cameras that are routinely used in MicroED were not designed for electron diffraction,^140^ so further hardware developments in this area must be made as well. Finally, high-throughput and automated approaches^141^ will be valuable to remove much of the expertise needed to conduct experiments to further democratize the approach. Finally, access to state-of-the-art equipment can be limiting for the application of MicroED. National centers, similar to what is available for tomography and SPA are need in order to reach the wider scientific community.

## FACILITIES FOR CRYO-EM USERS

One of the key difficulties that the field of cryo-EM has faced is ensuring access to the high-end and expensive electron microscopy hardware required to perform the various cryo-EM methods.

In the United States, the NIH has opened national centers for cryo-EM focusing on SPA, and later this was expanded to cryo-ET. These centers provide users access to electron microscopes for data collection and provide training to use the microscopes, all free of charge. The centers have developed a robust curriculum to give users hands on training in sample preparation and data collection, interpretation, and analysis. There are currently three national centers for cryo-EM in the USA—National Center for CryoEM Access & Training (NCCAT), Stanford-SLAC Cryo-EM Center (S2C2), and The Pacific Northwest Center for Cryo-EM (PNCC).

The national centers for Cryo-ET were founded later, in 2020, as a part of the transformative high-resolution Cryo-Electron Microscopy Program. The Midwest center for Cryo-ET, located in the University of Wisconsin Madison, acts the network hub. They provide access for academic and commercial data collection. Users can get trained on all the steps in the cryo-ET technique in this facility. The other three centers, CU Boulder Center for Cryo-ET (CCET), National center for *In situ* Tomographic Ultramicroscopy (NCITU), and Stanford-SLAC Cryo-ET Specimen preparation Center (SCSC), are responsible for sample preparation and screening.

In Europe, the national centers for cryo-EM include the Danish cryo-EM national facility in Denmark, Ernst Ruska-Center for Microscopy and Spectroscopy with Electrons (ER-C) in Germany, SOLARIS National Synchrotron Radiation Center in Poland, CryoEM CNB-CSIC Facility in Spain, and the National Facility for Structural Biology at Human Technopole in Italy. Another notable cryo-EM center is the National Center for Protein Science Shanghai (NCPSS) in China.

In contrast, there are currently no national centers for MicroED yet, which puts restraints on the use of this technique by researchers who do not have access to the expertise or instrumentation required to perform electron diffraction experiments.

## Data Availability

Data sharing is not applicable to this article as no new data were created or analyzed in this study.
